# Challenges and Recommendations for Proxy Reporting in Aging Research: A Brief Commentary

**DOI:** 10.7759/cureus.76587

**Published:** 2024-12-29

**Authors:** Selina Baidoo, Ojonimi S Salihu, Ejura Y Salihu

**Affiliations:** 1 Department of Sociology, Western Michigan University, Kalamazoo, USA; 2 Department of Family Medicine and Community Health, University of Wisconsin-Madison, Madison, USA

**Keywords:** aging research, older adults, proxies, proxy reporting, research participation

## Abstract

The US has an aging population that is under-represented in research. Many older adults face barriers to research participation, such as mobility issues, comorbidities, and declining physical and cognitive health, which make it harder for them to understand study processes and give informed consent. Proxies can be family members, paid or unpaid caregivers, or healthcare providers who provide health information for older adults. Proxy reporting is an important resource in aging research, but it is fraught with several challenges that can impact data accuracy and validity. In this paper, we describe these challenges and possible solutions to enhance proxy reporting and the participation of older adults in aging research.

## Introduction and background

The US has a rapidly aging population, with one in six adults being 65 years old or older. Currently, 18% of the US population are older adults, and this number is expected to increase to 23% by 2054 [1,2]. Despite the rising number of older adults in the US, this population remains underserved and hard to reach [3]. Several barriers to research participation and completion have been noted in this group. Some of these barriers include mobility issues, lack of transportation, and declining physical and cognitive health [4-6]. Declining cognitive health can be particularly challenging for older adults and makes it harder for them to understand study processes and give informed consent [7,8]. These barriers to participation make it difficult to recruit and retain older adults in research studies [3,5,7-9].

Proxy reporting is a low-to-no-cost, accessible, and sustainable way to involve older adults in research [8,9]. Proxies, who can be family members, paid or unpaid caregivers, or healthcare providers, provide health information on behalf of the older adult. Many older adults rely on proxies in various capacities, from participating in research to navigating public systems such as healthcare, legal, and social services [10,11]. Proxy reporting is a vital component of health research, offering an alternative way to understand the health status of elderly individuals, particularly those facing cognitive impairment, linguistic challenges, and comorbidities that hinder their direct participation in research [12-15].

Despite its significance, proxy reporting has several limitations and challenges, which can adversely affect research data and findings [14-17]. It's important to acknowledge that a one-size-fits-all approach may not be effective, and tailored strategies are needed to address the unique challenges of proxy reporting. Many studies that address the challenges with proxy reporting focus on other populations like children and teens [18-20] and proxy use in a singular context such as dementia or aphasia research [12,21,22], so they often do not adequately address the myriad of challenges that researchers using proxy reports face in different settings. This paper summarizes the literature on proxy reporting, provides an overview of a wide range of challenges with proxy reporting, specifically in the older adult population, and presents possible solutions and examples that might enhance the quality of proxy reports and improve research participation among older adults. This paper can serve as a springboard for further discourse on the challenges of proxy reporting and the development of a multi-pronged intervention that can improve the proxy reporting process and outcomes in this population.

## Review

Challenges with proxy reporting

One of the biggest challenges with proxy reports is the discordance between proxy reports and self-reports, which negatively impacts the accuracy and reliability of proxy reports [23-27]. The discordance and inaccurate reporting have been linked to the following factors:

Self-Schemas

One reason for the discordance is a phenomenon known as "self-schemas." Self-schemas are beliefs a person holds that influence their meaning-making and response to life events. A proxy's subconscious beliefs towards the health condition of older adults or even towards aging might affect how they view things, thus influencing their response and affecting data reliability. In this case, proxies might "reorganize or ascribe meanings to events" to accommodate their beliefs and expectations. Proxies may, therefore, not report how things really are but may unintentionally say what best suits their beliefs, which could lead to downplaying the conditions of older adults. Another challenge with proxy reporting related to self-schemas is the impact of proxy perception of self on their reports. Study findings showed that caregivers who rate themselves as highly stressed reported lower scores on health indicators in their proxy reports when compared to the patient's self-reports. Caregiver proxies who see themselves as good caregivers are more likely to provide responses affirming that belief, neglecting anything they think may jeopardize their identity as good caregivers. On the other hand, a caregiver who sees their job as tedious and something that prevents them from fulfilling other aspects of their lives may exaggerate conditions than they are to support their belief that they are working overly hard to the extent that they forgo their aspirations to take care of others [28,29].

Social Bias

Another challenge with proxy reports noted in literature is the tendency for social desirability bias. A study by Hack et al. showed that the relationship between the subject and the proxy influences proxy responses, such that there were differences in proxy reports by family caregivers and formal carers in nursing homes. Family carers shared more intimate relationships with subjects and were better positioned to answer questions concerning their older adults' well-being than formal carers [29]. This finding was validated by a similar study, which added that these family members also tended to provide more socially desirable information, perhaps in an attempt to portray a more favorable image of their family [28].

Inappropriate Measures for Proxy Reporting

Neumann and colleagues noted that an often-overlooked area of proxy reporting that can cause inaccuracies and challenge the reliability and validity of proxy reports is the construction of items for proxy ratings. Despite conceptual consistency among items on proxy questionnaires, it may still be challenging for proxies to respond appropriately. This becomes a challenge for proxies when these concepts and theories do not describe overt characteristics of patients but rather covert characteristics that proxies cannot observe directly. For instance, when questions center on covert characteristics such as emotions (like happiness or sadness), feelings (like loneliness or contentment), attitudes (like optimism or pessimism), and things done alone by the patient, it could be challenging for a proxy to answer such questions accurately. The authors found that these inappropriate measures could result in discrepancies in their reports when proxies cannot report on things that have subjective meanings [30].

Skewed Reporting Based on Patient’s Health Status

The reliability and validity of proxy reporting could also be influenced by the health status of the older adult. To understand how patient status played a role, a group of researchers conducted a Medline search from 1990 to 1999 for keywords such as "proxy" and "informant" in studies centered on people aged 60 and above. From their search, they found 24 relevant articles for analysis. Proxies provided more information on the physical and instrumental functionality of older people, with little coverage of areas related to cognition, physical health, medical care utilization, and overall quality of life. Additionally, when investigators conducted actual performance assessments, proxies tended to overestimate disabilities like walking and dressing while underreporting activities such as making phone calls, taking medication, and bathing [30]. Another study found that concordance was significantly higher between older adults and their proxies when the older adults had multi-morbidity (the presence of two or more chronic diseases or conditions) and pain [13].

High Non-response Rate Depending on the Proxy’s Relationship to the Older Adult

Wagner and colleagues conducted a study that showed that a high item non-response rate is a challenge in proxy reporting [9]. This challenge was noted in a 2000 study on functional abilities and continence among older people [31]. The authors noticed that with questions related to the frequency and severity of disease symptoms among older adults, there were more non-responses from proxies compared to when the question required a yes/no answer. They noted that the more private and less observable the item being measured is, the more likely they are not to receive a response from a proxy. To further understand the challenge of proxy item non-response, the researchers conducted a study with women aged 65 years and older who underwent surgery for a fractured femur to determine their functional ability and continence. To ensure the percentage of non-response items, similar interview questions were administered by trained interviewers to both the patients and selected proxies. The majority of the questions related to functional ability had non-response from proxies. The authors suggested the high rate of no response may be attributed to the fact that only 32.1% of proxy respondents lived with the subjects, while 74.7% did not live with the subjects and only visited daily or two to three times per week. This result is validated by other studies that showed that when proxies do not observe the person frequently to know their various activities, it reduces the level of familiarity needed to answer questions about frequency and the likelihood of item non-response [9,28].

Lack of Culturally Tailored Measures

Many questionnaires being used for proxy reporting are not culturally appropriate for different cultural contexts, especially communities of color. When culturally inappropriate proxy questionnaires are used, the quality of data collected may be compromised because each proxy has different capabilities, cultural beliefs, educational attainment, and socio-economic class that influence how they interpret and respond to questions [28]. Specifically, cultural differences such as language and perceptions about certain health issues may influence the responses of these proxies.

Recommendations and examples for implementing these recommendations in different settings

Proxies are essential sources of health information for older adults, but when they are unable to provide accurate information, they can be misleading, and researchers may not be well-informed enough to make concrete conclusions. Researchers can avoid some of the pitfalls with proxy reports by incorporating some of these recommendations in their work.

Consideration of Proxy’s Ability and Role in the Subject’s Life

To enhance the reliability and validity of proxy reports, it is important to identify proxies with adequate or relevant information early on in the research process and have them follow the older adult throughout the study. When assessing a patient's health status, a neighbor might have limited knowledge about specific relevant details compared to a spouse or close family member involved in daily care routines. Therefore, selecting a proxy who is more familiar with the subject is essential, such as someone who regularly monitors the patient's medication or symptom management. In a study on elderly care, caregivers (such as family members) may fill out questionnaires about the patient's daily functioning, while healthcare professionals might be asked to rate the patient's medical status or health outcomes. Also, if the study focuses on mental health outcomes for older adults, the selected proxy could be a caregiver or spouse who is familiar with their behavior and mental state. However, if the study focuses on social skills, neighbors, colleagues, or friends might be more appropriate proxies because of their direct involvement in the subject's social life.

Some researchers recommend using different proxies to assess different aspects of the quality of life for older people to address some of the challenges with proxy reporting. A 2022 study showed that using clinicians to answer questions on the physical abilities of older people proved more effective through their use of what they called the Bristol Activity of Daily Living Scale because clinicians' responses were more closely associated with functional ability. They also found that live-in family members had responses that were more closely related to psychosocial and behavioral symptoms, as measured by the Neuropsychiatric Inventory [27]. This recommendation can be incredibly beneficial in improving proxy reporting, as proxies may be more knowledgeable in certain areas of the respondents' lives than others.

Give Room for Admitting Lack of Knowledge in Survey Responses

Incompatible proxy ratings lead to inaccurate proxy responses on the assessment of a patient's health [28]. There is a need to improve proxy respondents' questionnaires by introducing "I don't know" options or answers to ensure that inaccurate responses are not given. One recommendation for navigating the challenges that proxies face when they are unfamiliar with the aspect of the respondents' lives is to include the option of admitting that they do not know the answer. By forcing proxies to answer yes or no responses, there is a tendency to give an inaccurate report. Researchers should also consider including open-ended questions so proxies can expand on their answers or provide a rationale for their answers (or lack thereof).

Complement Subjective Proxy Reports With Objective Measures

Another recommendation is for researchers to complement proxy reports with objective measures such as medical records and claims data, where inaccuracies in reports can be easily detected [30]. Aging researchers should endeavor to establish a strong correlation between proxy reports and objective measures to ensure that reports from proxies represent the actual state or condition of the health status of older people being studied. Familiarizing oneself with the symptoms of common age-related diseases, such as Alzheimer's, can help researchers compare symptoms being shared by proxies with the knowledge they have also acquired from their study on that health condition. This helps detect discrepancies in proxy reports and helps researchers keep more reliable data to make informed decisions. In a study on chronic disease management, proxy reports from caregivers can be cross-checked with objective data like hospital records, prescription history, or insurance claims, to validate the accuracy of reported health conditions.

Longitudinal Studies Using Validated Measures to Track Changes in the Proxy Report

Longitudinal studies can be particularly useful for understanding how proxy reports evolve, which can help researchers monitor trends and notice potential biases and errors in these reports. By collecting data from proxies at multiple time points, it is easier to notice patterns of change in proxy reports versus the results of objective measures. This can also help researchers identify factors that impact proxy accuracy and evaluate whether proxies provide consistent responses that align with changes in the subject's condition or circumstances [30].

Use of Validated Proxy Measures

Some studies have also shown that proxy reports may not be suitable for some measures [23]. To address this challenge, measures used for proxy reports should be specially designed for proxies and validated using gold-standard benchmarks. While survey validation can be time-consuming, using measures that have yet to be validated can introduce biases and compromise the reliability of findings, exacerbating the risks associated with proxy reports. Special care should be taken to ensure that proxy measures are appropriate, reliable, and valid to maintain the research's integrity and the reliability of findings [23].

By employing these methods, researchers could improve the reliability and validity of proxy reports in assessing the health status of older populations where direct assessment and self-reports are not feasible.

## Conclusions

Proxies are important in aging research because they make it possible to include individuals who would have otherwise not been included. The use of proxies is also likely to become more common as rates of Alzheimer's disease and other cognitive impairments increase. As new drugs and new treatment methods are approved, proxies are increasingly relied upon to determine the quality of life of these patients and improve their health. Although proxy reports are not generalizable and often fraught with challenges such as discordance and the use of inappropriate measures, proxies are still a valuable source of information that helps to shape research agendas and interventions to improve the health outcomes of older adults. There is an urgent need for tailored approaches that incorporate these recommendations to address the challenges of proxy reporting and improve the proxy reporting process. Such efforts can support and sustain accurate proxy assessments, ensuring the continued participation of older adults in research.
